# Validation and Application of Functions of Future Thinking Scale in Chinese Adults

**DOI:** 10.1002/pchj.819

**Published:** 2024-12-08

**Authors:** Lulu Liu, Lijuan Dai, Ya Wang

**Affiliations:** ^1^ Department of Psychology, Faculty of Social Sciences University of Macau Macao China; ^2^ Centre for Cognitive and Brain Sciences, Institute of Collaborative Innovation, University of Macau Macao China; ^3^ School of Psychology, Capital Normal University Beijing China

**Keywords:** adaptive behavior, confirmatory factor analysis, emotional states, future thinking, validation

## Abstract

Future thinking, mentally projecting oneself into future events, scenarios, and circumstances, is common in everyday life. However, no scale has been developed to explore the functions of future thinking in China. This study aimed to validate the Chinese version of the functions of future thinking scale (FoFTS). Based on a sample of 578 Chinese residents, confirmatory factor analysis results indicated that the 10‐factor structure of the Chinese version of FoFTS fit well. The reliability indexes across 10 factors were in an acceptable range. Acceptable convergent validity was reported considering its association with time perspective, future self‐continuity, emotion regulation, and intertemporal decision‐making. Additionally, the effect of age and the severity of emotional states on FoFTS were found. Overall, the Chinese FoFTS is a reliable and valid tool for examining the diverse purposes and roles of future thinking among Chinese adults, thereby enhancing the cross‐cultural study of purposes for future thinking.

## 1 Introduction

Future thinking refers to the ability to project oneself to the future and pre‐experience events (Atance and O'Neill 2001). Thinking about the future is typical behavior in humans, from ancient times, when people prepared for life‐threatening events, to the present day, when we plan our everyday activities. Researchers have proposed a framework of future thinking that includes four modes: simulating a detailed future scenario, predicting the consequences and possible reactions to events, encoding future intentions, and planning steps to achieve future goals (Szpunar, Spreng, and Schacter 2014). Moreover, future thinking is relatively frequent and positivity‐biased in our daily lives (Barsics, Van der Linden, and D'Argembeau 2016); for instance, many individuals exhibit an overly optimistic outlook on their personal future.

Previous theoretical and empirical research has shed light on the benefits of future thinking by examining its connection with adaptive cognitions and behaviors. Future thinking is a crucial cognitive function that serves a wide range of functions, such as emotion regulation (Barsics, Van der Linden, and D'Argembeau 2016), prospective memory (Schacter, Benoit, and Szpunar 2017), sense of identity (D'Argembeau and Garcia Jimenez 2023), and farsighted decision‐making (Rosch, Stramaccia, and Benoit 2022). Furthermore, researchers have suggested that future thinking is linked to intertemporal decision‐making (Schacter, Benoit, and Szpunar 2017) and can positively improve delay discounting (Ciaramelli et al. 2021; Palombo, Keane, and Verfaellie 2016). While many previous studies have focused on the effects of future thinking, it is equally important to understand the processes of future thinking and the use of future thinking for a prescribed purpose. From a functional perspective, exploring the purposes of future thinking can clarify the differences between “future thinking” and the more general tendency to “think about the future” (time perspective), as well as the connection between a person's current self and future self (present–future self‐continuity). Furthermore, it predicts the relationships among future thinking, emotional states (for instance, depression and anxiety), and behavioral outcomes (such as intertemporal choice).

Adopting a functional approach to future thinking, researchers developed and validated a standardized measure for self‐reported functions of future thinking, the Functions of Future Thinking Scale (FoFTS), in a sample of the Australian population (Hallford and D'Argembeau 2021). This scale assessed ten functions of future thinking, i.e., boredom reduction, death preparation, identity contrast, negative emotion regulation, positive emotion regulation, social bonding, goal setting, planning, problem solving, and decision making. This 30‐item scale, with good reliability and validity, provided a standardized tool for assessing why people engage in the future in their everyday lives. To date, a Persian version of the FoFTS has been developed and validated (Akbari et al. 2023) from the original English version. In contrast, it has yet to be validated in China to explore the functions of future thinking.

Since culture and social environments shape our future thinking, a validated Chinese version of FoFTS is necessary to explore its purposes and functions within the Chinese context. Researchers identified cultural and environmental differences in investigating functions and content of future thinking (Mert, Hou, and Wang 2023; Özbek, Bohn, and Berntsen 2020). For instance, Chinese people value a past event over an identical future event, while Canadians reported the opposite (Guo et al. 2012). A comparative study on cultural differences between Chinese and Australian university students suggested that Australians imagined more achievement and life‐threatening events in the future than their Chinese counterparts. In contrast, Chinese students imagined more about careers in the future (Chen et al. 2015). These empirical studies demonstrated that individuals from diverse cultures emphasize different aspects of daily life and consider distinct elements of their future. This results in unique attitudes and behaviors, such as time perspective, self‐concept, and intertemporal decision‐making.

The way an individual thinks about the future is not only shaped by cultural and social environments but also by individual differences, such as ageing (Rendell et al. 2012), depression, anxiety, and stress states (Seli et al. 2019). Research has shown that younger adults reported more engagement in future thinking than older adults, and thinking about the future moderates the association between higher arousal of positive affect and everyday happiness in older adults (Choi et al. 2024). Phenomenologically, younger adults are more sensitive to goal activation of future thinking than older adults (Lapp and Spaniol 2017). As such, questions related to the age are still unresolved. For instance, do different age groups think about the future with distinct purposes, and if so, which purposes do various age groups hold onto?

Moreover, depression, anxiety, and stress are closely related to increased thoughts about the past, present, and future (Seli et al. 2019). People who scored higher in depression generated fewer positive future events than those with lower scores (Kosnes et al. 2013). Individuals with depression often show deficits in episodic future thinking (Hallford et al. 2018). Likewise, future thoughts have also been suggested to be associated with stress (Branch 2023). It would be interesting to explore whether FoFTS can distinguish people with different levels of depression, anxiety, and stress. By comprehending the functions of future thinking, we can effectively utilize future thinking as a promising intervention for individuals with depression, anxiety, and stress states (DASS) or symptoms.

The current study aimed to validate the Chinese version of FoFTS and investigate its application in Chinese adults. Specifically, we hypothesized that the structure and psychometric properties of the Chinese FoFTS were similar to those of its English version, displaying acceptable reliability and validity. This study also sought to explore age differences in functions of future thinking among Chinese adults, with the hypothesis that functions of future thinking change with ageing. Additionally, the study compared the functions of future thinking across DASS to investigate the discriminative application of the Chinese FoFTS. We hypothesized that the Chinese FoFTS could be used effectively to differentiate between different levels of DASS.

## 2 Methods

### 2.1 Participants

Chinese adult residents from different cities were randomly recruited online. The inclusion criteria were age over 18 and under 60, being a native Chinese speaker, and providing complete answers to all the measures. Using these criteria, 606 participants were recruited in this study. Eleven participants who reported unmatched age and education have been excluded. Seventeen participants whose response time was too long or short (three standard deviations over/below the mean response time) were identified as invalid data and excluded from the statistical analyses. A total of 578 participants were included in the current study.

### 2.2 Procedures

The present study was conducted in accordance with the Declaration of Helsinki and was approved by the ethics committee of University of Macau. Before collecting the data, we obtained permission from the first author of FoFTS to use and revise the questionnaire (Hallford and D'Argembeau 2021). The translation followed international norms (Sousa and Rojjanasrirat 2011). The original English version of the scale was forward‐translated into simplified Chinese by two independent professional translators fluent in English and Chinese. The two forward‐translated versions of the scale and the preliminary initial translated version were compared by a third professional translator, then the three translators and the investigator formed a committee. After that, the preliminary initial version was back‐translated into English by two other doctoral students who were blind to the original English version of FoFTS. Comparisons were conducted between the original and translated versions and two back‐translated versions. The committee has discussed and resolved ambiguities and discrepancies to ensure the accuracy of the content.

The study was conducted online from October 2023 to January 2024. The sample size was established using a sample‐to‐variable ratio of 10:1, not below 100 participants (Nunnally 1994), where the variable was defined as the item. Therefore, a minimum of 300 participants should be gathered. To ensure the stability of the results, as much data as possible with the funding allowed was collected. Participants were recruited from an online survey provider (Credamo; www.credamo.com), a Chinese platform for questionnaire data collection. The purpose of the study was explained before the survey. Participants' informed consent was obtained by answering one question at the beginning of the study. Participants were compensated with 10 Chinese Yuan (CNY) for completing the survey.

### 2.3 Materials

#### 2.3.1 Functions of Future Thinking Scale

This 30‐item scale measured 10 different functions of future thinking: planning, goal setting, problem solving, decision making, social bonding, upregulating positive emotion, downregulating negative emotion about future experiences, reducing boredom, thinking about the type of person one wants to become (identity contrast), and think about and adjusting to death (death preparation) (FoFTS; Hallford and D'Argembeau 2021). Participants were asked to report why they think about their own future about events that might personally happen to them. Each function includes three items, scored on a 5‐point Likert scale from 1 (*never*) to 5 (*very often*). The range of total scores for each function was from 3 to 15, with higher scores indicating more use of future thinking for that function. Internal consistency for the 10 factors ranges from 0.78 to 0.91.

#### 2.3.2 Future Self‐Continuity Questionnaire

This 10‐item questionnaire examined present‐to‐future self‐continuity (FSCQ; Shen, Wang, and Zhou 2022; Sokol and Serper 2020). Participants were required to rate their states of the future self in terms of similarity, vividness, and positive affect to the present self. Each item was rated on a 6‐point Likert scale from 1 to 6. Higher scores indicated higher levels of present‐to‐future self‐continuity. The Cronbach's alpha coefficient for the total score was 0.78.

#### 2.3.3 Emotion Regulation

The 10‐item questionnaire measured emotion regulation strategies from two dimensions: cognitive reappraisal and expression suppression (ERQ; Gross and John 2003; Luo et al. 2012). Items were rated on a 7‐point Likert scale from 1 (*totally disagree*) to 7 (*totally agree*), asking participants about their emotional regulatory process. The internal consistency for cognitive reappraisal and expression suppression were 0.80 and 0.79, respectively.

#### 2.3.4 Zimbardo Time Perspective Inventory

This inventory was a 20‐item short Chinese version measuring people's time perspective from five dimensions: past‐positive, past‐negative, present‐hedonistic, present‐fatalistic, and future (ZTPI; Wang et al. 2015). All items for each dimension were scored on a 5‐point Likert scale ranging from 1 (*very uncharacteristic*) to 5 (*very characteristic*). The total score for each dimension ranged from 4 to 20. The internal consistency for the five dimensions ranges from 0.61 to 0.71.

#### 2.3.5 Monetary Choice Questionnaire

The 27‐item questionnaire assessed intertemporal decision‐making (MCQ; Kirby and Marakovic 1996). Participants were required to make decisions, choosing between smaller immediate rewards and larger delayed rewards. The delayed durations varied from 1 week to about 6 months. People tend to decrease the value of the future reward, which is delay discounting. The delay discounting rate (*k*) was calculated using the hyperbolic model to reflect participants' intertemporal decision making, with a higher score indicating a higher tendency to choose the smaller immediate choice. A natural log (ln) function has been used to transform all the non‐normal distributed *k* values for further analysis.

#### 2.3.6 Depression, Anxiety, and Stress Scale

This short‐form scale was used to measure participants' depression, anxiety, and stress states (DASS‐21; Gong et al. 2010; Henry and Crawford 2005). Each subscale is measured by seven items with a range from 0 to 42 (the summed score is multiplied by 2), with higher scores indicating increased levels of severity. For the depression dimension, a score ranging from 0 to 13 indicates normal to mild severity, and a score over 14 indicates moderate to extreme severity. For the anxiety dimension, a score ranging from 0 to 9 indicates normal to mild severity, and a score over 10 indicates moderate to extreme severity. For the stress dimension, a score ranging from 0 to 18 indicates normal to mild severity, and a score over 19 indicates moderate to extreme severity. The internal consistency of the Chinese version for depression, anxiety, and stress were 0.77, 0.79, and 0.76, respectively.

#### 2.3.7 Frequency of Near‐ and Far‐ Future Thinking

Previous researchers proposed that people's general tendency to think about the future would influence their propositions to the functions of future thinking. Hallford and D'Argembeau (2021) adopted six items to assess people's frequency of thinking about the near and far future. All six items started with the statement, “How frequently do you tend to think about your future for things that will happen …” with the near‐future thinking containing branches “later that day, tomorrow, over the next week,” and with the far‐future thinking including branches “over the next year, in over a year, over ten years.” Questions were scored on a 5‐point Likert scale from 0 (*never*) to 4 (*most of the time*), and the range of scores for near‐ and far‐ future thinking was from 0 to 12. A higher score means a stronger tendency to think about the near‐ or far‐ future. The Cronbach's alpha coefficients for near‐ and far‐future thinking were 0.73 and 0.74, respectively.

### 2.4 Data Analysis

The data were analyzed using R package version 4.3.0 and AMOS version 25. The normal distribution of the data for each item has been checked using skewness (−1.80–1.20) and kurtosis coefficient (−0.28–1.00) (Yuan and Bentler 2001). The descriptive statistics were first reported. Since the factor structure of English‐version FoFTS has been validated, the validity of the factor structure in Chinese participants was assessed using confirmatory factor analysis (CFA). A variety of indices were used to evaluate the model fit, including comparative fit index (CFI), Tucker–Lewis Index (TLI), root mean square error of approximation (RMSEA), and standardized root mean square residual (SRMR). An acceptable fit requires the CFI and TLI values greater than 0.90 and the RMSEA and SRMR less than 0.80 (McDonald and Ho 2002). Internal reliability was assessed using Cronbach's alpha coefficient. Convergent validity was evaluated based on the correlations across FoFTS subscales and between FoFTS subscales and other validity scales, that is, time perspective, future self‐continuity, emotion regulation, and intertemporal decision‐making. Given the number of correlations and inflated risk of Type I error, the alpha level was set at 0.001 for these analyses. Meanwhile, incremental validity informs us about the significance of the new measure in understanding emotional states and behavioral outcomes, above and beyond existing measures (Hunsley and Meyer 2003). Hierarchical multiple regression analyses were performed to assess whether FoFTS predict unique variance in future self‐continuity, emotion regulation, time perspective, delay discounting, and the severity of depression, anxiety, and stress related to the frequency of near‐ and far‐ future thinking.

For the application of FoFTS in Chinese adults, to compare the scores across FoFTS subscales in Chinese adults, a repeated measures ANOVA was conducted with Bonferroni correction for pairwise contrasts and Cohen's *d* as effect sizes. To examine the effects of demographics (sex, education, and age) on FoFTS subscales, an independent *t* test was used for sex, and Pearson correlations were conducted for education and age. To further explore age differences across adulthood, we divided participants into two groups: younger adults (*n* = 283, age range: 18–30) and middle‐aged adults (*n* = 295, age range: 31–60). Multivariate analysis with education as a covariate was used to assess for age differences in scores on FoFTS subscales. Finally, three multivariate analyses of covariance (MANCOVA) with age and education as covariates were used to explore the functions of future thinking across the severity of depression, anxiety, and stress.

## 3 Results

### 3.1 Participant Characteristics

Table 1 shows the demographic characteristics of the sample. The sample consisted of 578 participants (18–59 years). Most had a university degree (14% high school, 84% university degree). The scores of each FoFTS subscale are also presented in Table 1. The boredom and death functions scored lower than the subscale's median score (9), and the other functions scored higher than the median score of 9.

**TABLE 1:** Demographic characteristic and subscale scores of the sample (*N* = 578).

Variables	Total sample	
	Mean	SD
Sex: female (*n*/%)	295	51.03
Age	31.81	8.51
Years of education	16.30	2.11
FoFTS subscales		
Boredom	7.48	2.60
Death	5.58	2.45
Identity	12.40	1.63
Negative regulation	10.60	2.28
Social bonding	11.40	2.58
Goal setting	12.59	1.79
Planning	12.33	1.80
Problem solving	12.42	1.73
Decision making	11.83	2.01
Positive regulation	12.18	1.95

### 3.2 Chinese FoFTS Structure Validity: CFA

**FIGURE 1:**
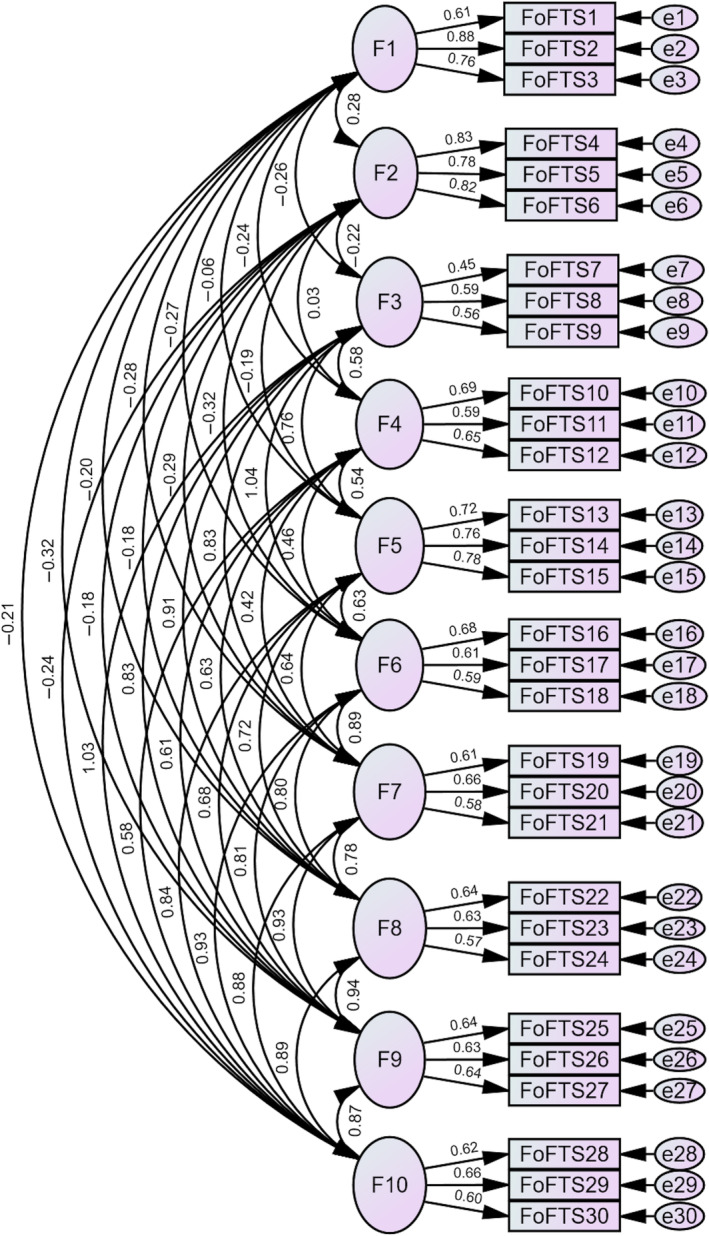
Chinese FoFTS structure with standardized estimates (*N* = 578). *Note:* F1, boredom reduction; F2, death preparation; F3, identity contrast; F4, negative emotion regulation; F5, social bonding; F6, goal setting; F7, planning; F8, problem solving; F9, decision making; F10, positive emotion regulation.

The Chinese FoFTS structure, depicted in Figure 1, was comparable to the proposed model in the initial validation study (Hallford and D'Argembeau 2021). The 10‐factor model with no cross‐loading or correlated residuals demonstrated good fit indices: *X* ^2^/*df* = 2.28, CFI = 0.93, TLI = 0.91, RMSEA = 0.05 [0.04, 0.05], and SRMR = 0.05. The item loadings were between 0.45 and 0.88. In contrast, the one‐factor model was considerably poorer: *X* ^2^/*df* = 6.41, CFI = 0.65, TLI = 0.62, RMSEA = 0.10 [0.09, 0.10], and SRMR = 0.09.

### 3.3 Chinese FoFTS Reliability

The Cronbach's alpha coefficients are summarized in Table 2. The Cronbach's alpha (*α*) for most subscales ranged from 0.64 to 0.85. The results showed acceptable internal consistency for Chinese FoFTS and indicated that it is a relatively reliable measurement. It should be noted that Cronbach's alpha for identity contrast is 0.55, which is relatively low. Thus, the result of this subscale identity contrast should be considered carefully.

**TABLE 2:** Reliability indices of the Chinese FoFTS.

	Cronbach's alpha
Boredom reduction	0.80
Death preparation	0.85
Identity contrast	0.55
Negative emotion regulation	0.68
Social bonding	0.80
Goal setting	0.66
Planning	0.64
Problem‐solving	0.65
Decision making	0.67
Positive emotion regulation	0.67

### 3.4 Convergent Validity

As shown in Table 3, the zero‐order correlations among the FoFTS subscales revealed a strong correlation among most factors. Additionally, the majority of results remained consistent in the partial correlation, even after adjusting for the frequency of near‐ and far‐future thinking. Separate analyses were performed to examine zero‐order correlations between the Chinese FoFTS subscales and validity scales, as well as partial correlations that account for the frequency of near‐ and far‐future thinking (see Table 4). Results showed that most Chinese FoFTS subscales, but not boredom reduction and death preparation, demonstrated positive associations with ZTPI past positive, ZTPI present hedonistic, ZTPI future, FSCQ future self‐continuity, and ERQ cognitive reappraisal. However, they were negatively correlated with ZTPI past negative and ZTPI present fatalistic. Additionally, ER emotion expressive suppression showed a positive correlation with negative emotion regulation and decision‐making functions in FoFTS. The MCQ delay discounting rate showed a negative association with boredom reduction and death preparation but a positive correlation with negative emotion regulation and decision‐making in FoFTS.

**TABLE 3:** Intercorrelations between the Chinese FoFTS subscales (Zero‐order correlation on the bottom left diagonal and partial correlation controlling for the frequency of near‐ and far‐ future thinking on the right diagonal).

	F1	F2	F3	F4	F5	F6	F7	F8	F9	F10
F1	—	0.10	−0.08	0.10	0.18	−0.17	−0.09	0.08	−0.06	0.02
F2	**0.23**	—	−0.09	0.20	−0.24	−0.23	−0.19	−0.04	−0.04	−0.16
F3	**−0.13**	**−0.12**	—	**0.36**	**0.33**	**0.60**	**0.45**	**0.53**	**0.44**	**0.60**
F4	**−0.18**	0.02	**0.36**	—	**0.27**	0.19	0.14	**0.41**	0.25	**0.38**
F5	−0.02	**−0.15**	**0.49**	**0.40**	—	**0.46**	**0.34**	**0.33**	**0.32**	**0.47**
F6	**−0.17**	**−0.24**	**0.63**	**0.31**	**0.46**	—	**0.68**	**0.51**	**0.54**	**0.65**
F7	**−0.17**	**−0.22**	**0.47**	**0.29**	**0.46**	**0.57**	—	**0.43**	**0.60**	**0.58**
F8	−0.11	**−0.13**	**0.54**	**0.42**	**0.51**	**0.53**	**0.50**	—	**0.56**	**0.54**
F9	**−0.21**	**−0.14**	**0.50**	**0.42**	**0.50**	**0.55**	**0.62**	**0.62**	—	**0.52**
F10	−0.10	**−0.17**	**0.60**	**0.40**	**0.61**	**0.61**	**0.57**	**0.58**	**0.58**	—

*Note:* All bolded correlations are *p* < 0.001. F1, boredom reduction; F2, death preparation; F3, identity contrast; F4, negative emotion regulation; F5, social bonding; F6, goal setting; F7, planning; F8, problem solving; F9, decision making; F10, positive emotion regulation.

**TABLE 4:** Pearson and partial correlation between Chinese FoFTS subscales and validity scales.

	ZTPI Past positive		ZTPI Past negative		ZTPI Present hedonistic		ZTPI Present fatalistic		ZTPI future		Future self‐continuity		ERQ Cognitive reappraisal		ERQ Expressive suppression		MCQ Delay discounting	
	r	$r_{p}$	r	$r_{p}$	r	$r_{p}$	r	$r_{p}$	r	$r_{p}$	r	$r_{p}$	r	$r_{p}$	r	$r_{p}$	r	$r_{p}$
Boredom reduction	**−0.14**	−0.06	**0.22**	**0.30**	0.00	−0.04	**0.14**	0.23	**−0.18**	−0.26	**−0.18**	−0.15	**−0.22**	**−0.29**	−0.09	−0.04	−0.13	0.08
Death preparation	**−0.14**	0.03	**0.15**	0.15	−0.06	−0.01	**0.21**	0.20	**−0.17**	−0.13	**−0.20**	−0.25	**−0.13**	−0.19	0.03	−0.12	**−0.17**	−0.20
Identity contrast	**0.39**	**0.29**	**−0.43**	**−0.37**	**0.39**	**0.53**	**−0.47**	**−0.46**	**0.50**	**0.53**	**0.52**	**0.52**	**0.51**	**0.56**	0.02	−0.08	−0.02	−0.11
Negative emotion regulation	**0.30**	0.22	**−0.21**	−0.17	**0.25**	**0.34**	**−0.16**	−0.07	**0.30**	0.21	**0.31**	0.16	**0.32**	0.20	**0.29**	−0.14	**0.20**	−0.09
Social bonding	**0.39**	**0.32**	**−0.37**	−0.22	**0.35**	**0.45**	**−0.36**	**−0.29**	**0.40**	**0.36**	**0.47**	**0.39**	**0.36**	0.23	**0.13**	−0.10	0.10	−0.14
Goal setting	**0.41**	**0.31**	**−0.36**	**−0.29**	**0.42**	**0.50**	**−0.51**	**−0.51**	**0.56**	**0.56**	**0.56**	**0.54**	**0.49**	**0.50**	0.04	0.00	0.04	−0.03
Planning	**0.43**	**0.43**	**−0.32**	−0.21	**0.29**	**0.37**	**−0.46**	**−0.42**	**0.52**	**0.51**	**0.47**	**0.51**	**0.46**	**0.48**	0.04	−0.08	0.10	0.08
Problem‐solving	**0.37**	**0.41**	**−0.32**	−0.18	**0.30**	**0.34**	**−0.35**	**−0.36**	**0.41**	**0.38**	**0.42**	**0.39**	**0.41**	**0.34**	0.11	0.01	0.13	0.04
Decision making	**0.37**	0.21	**−0.37**	−0.21	**0.29**	**0.32**	**−0.41**	**−0.32**	**0.42**	**0.31**	**0.49**	**0.36**	**0.41**	**0.29**	**0.14**	0.01	**0.13**	0.04
Positive emotion regulation	**0.50**	**0.37**	**−0.41**	**−0.39**	**0.38**	**0.52**	**−0.50**	**−0.47**	**0.50**	**0.53**	**0.58**	**0.57**	**0.48**	**0.52**	0.05	−0.11	0.07	−0.01

*Note:* All bolded correlations are *p* < 0.001. $r_{p}$: Controlled partial correlations for frequency of near‐ and far‐ future thinking.

Abbreviations: MCQ, monetary choice questionnaire; ZTPI, zimbardo time perspective inventory.

### 3.5 Incremental Validity

The results of multiple linear regression are shown in Table 5. For FSCQ future self‐continuity, the FoFTS subscales explained a total variance of 0.43, with unique variance predicted by identity contrast, social bonding, goal setting, decision‐making, and positive emotion regulation. For ERQ cognitive reappraisal, the FoFTS subscales explained a total variance of 0.35, with unique variance predicted by boredom reduction, identity contrast, negative emotion regulation, goal setting, planning, and positive emotion regulation. For ERQ expressive suppression, the FoFTS subscales explained a relatively low variance of 0.09, with unique variance predicted by negative emotion regulation. For ZTPI past‐positive perspective, the FoFTS subscales explained a total variance of 0.29, with unique variance predicted by planning and positive emotion regulation. For ZTPI past‐negative perspective, the FoFTS subscales explained a total variance of 0.26, with unique variance predicted by boredom reduction, identity contrast, social bonding, and positive emotion regulation. From ZTPI present‐hedonistic perspective, the FoFTS subscales explained a total variance of 0.22, with unique variance predicted by boredom reduction, identity contrast, social bonding, and goal setting. From ZTPI present‐fatalistic perspective, the FoFTS subscales explained a total variance of 0.35, with unique variance predicted by identity contrast, negative emotion regulation, goal setting, planning, and positive emotion regulation. For ZTPI future perspective, the FoFTS subscales explained a total variance of 0.40, with unique variance predicted by identity contrast, goal setting, and planning. For MCQ delay discounting, the FoFTS subscales explained a variance of 0.09, with unique variance predicted by death preparation, identity contrast, and negative emotion regulation. For DASS depression, the FoFTS subscales explained a total variance of 0.30, with unique variance predicted by boredom reduction, death preparation, identity contrast, negative emotion regulation, goal setting, planning, decision‐making, and positive emotion regulation. For DASS anxiety, the FoFTS subscales explained a total variance of 0.21, with unique variance predicted by boredom reduction, death preparation, and planning. For DASS stress, the FoFTS subscales explained a total variance of 0.26, with unique variance predicted by boredom reduction, death preparation, negative emotion regulation, social bonding, and planning.

**TABLE 5:** Incremental validity of the Chinese FoFTS.

Variable	Future self‐continuity		ERQ Cognitive reappraisal		ERQ Expressive suppression		ZTPI Past‐positive		ZTPI Past‐negative		ZTPI Present‐hedonistic		ZTPI Present‐fatalistic		ZTPI Future		MCQ Delay discounting	
	*β*	t	*β*	t	*β*	t	*β*	t	*β*	t	*β*	t	*β*	t	*β*	t	*β*	t
Boredom reduction	−0.07	−1.94	*−0.11*	*−3.11*	−0.05	−1.23	−0.06	−1.46	***0.15***	***3.91***	**0.08**	**2.06**	0.04	0.99	−0.06	−1.83	−0.06	−1.44
Death preparation	−0.05	−1.47	0.01	0.23	0.02	0.53	−0.02	−0.47	0.03	0.72	0.02	0.45	0.07	1.81	−0.01	−0.37	***−0.17***	***−3.89***
Identity contrast	***0.14***	***3.23***	***0.22***	***4.60***	−0.11	−1.88	0.04	0.82	***−0.23***	***−4.55***	**0.13**	**2.49**	***−0.17***	***−3.50***	***0.15***	***3.32***	*−0.18*	*−3.15*
Negative emotion regulation	0.01	0.37	**0.08**	**2.01**	***0.28***	***5.96***	0.08	1.92	0.05	1.16	0.08	1.85	*0.11*	*2.78*	0.05	1.29	***0.21***	***4.39***
Social bonding	**0.11**	**2.50**	0.00	0.07	0.10	1.84	0.08	1.77	***−0.16***	***−3.22***	**0.11**	**2.24**	−0.03	−0.73	0.05	1.14	0.03	0.62
Goal setting	***0.19***	***4.15***	*0.13*	*2.63*	0.00	−0.07	0.07	1.40	−0.01	−0.20	***0.25***	***4.60***	***−0.18***	***−3.69***	***0.25***	***5.27***	−0.06	−1.04
Planning	0.05	1.22	***0.16***	***3.45***	−0.05	−0.94	***0.17***	***3.51***	0.00	0.09	0.00	−0.04	*−0.14*	*−2.88*	***0.23***	***5.16***	0.03	0.53
Problem solving	−0.06	−1.34	0.03	0.62	0.01	0.18	0.03	0.53	0.03	0.52	−0.02	−0.28	0.04	0.82	0.00	0.03	0.08	1.41
Decision making	**0.11**	**2.34**	−0.02	−0.30	0.10	1.58	−0.05	−0.96	−0.10	−1.88	−0.01	−0.24	−0.06	−1.21	−0.04	−0.89	0.06	1.03
Positive emotion regulation	***0.22***	***4.59***	**0.13**	**2.42**	−0.09	−1.37	***0.25***	***4.59***	**−0.13**	**−2.24**	0.07	1.29	***−0.21***	***−3.93***	0.08	1.60	−0.02	−0.30
	R ^2^ = 0.43		R ^2^ = 0.35		R ^2^ = 0.09		R ^2^ = 0.29		R ^2^ = 0.26		R ^2^ = 0.22		R ^2^ = 0.35		R ^2^ = 0.40		R ^2^ = 0.09	

Variable	DASS Depression		DASS Anxiety		DASS Stress	
	β	t	β	t	β	t
Boredom reduction	*0.11*	*2.94*	***0.17***	***4.27***	***0.28***	***7.27***
Death preparation	***0.15***	***4.12***	**0.09**	**2.32**	*0.10*	*2.68*
Identity contrast	*−0.15*	*−3.00*	−0.06	−1.20	−0.02	−0.31
Negative emotion regulation	*0.12*	*2.80*	0.04	0.86	**0.09**	**2.04**
Social bonding	−0.07	−1.40	−0.09	−1.73	***−0.19***	***−3.84***
Goal setting	***−0.25***	***−4.87***	−0.09	−1.69	−0.03	−0.49
Planning	**−0.13**	**−2.56**	***−0.22***	***−4.18***	***−0.20***	***−3.86***
Problem solving	−0.02	−0.34	−0.03	−0.59	−0.02	−0.39
Decision making	*0.14*	*2.69*	0.07	1.22	−0.01	−0.20
Positive emotion regulation	**−0.11**	**−2.05**	−0.06	−1.02	−0.02	−0.35
	R ^2^ = 0.30		R ^2^ = 0.21		R ^2^ = 0.26	

*Note: R* is in adjusted form. Bold values indicate significance at the *p* < 0.05 level. Italic values indicate significance at the *p* < 0.01 level. Bold and italic values indicate significance at the *p* < 0.001 level.

### 3.6 Chinese FoFTS Subscales Comparison

**FIGURE 2:**
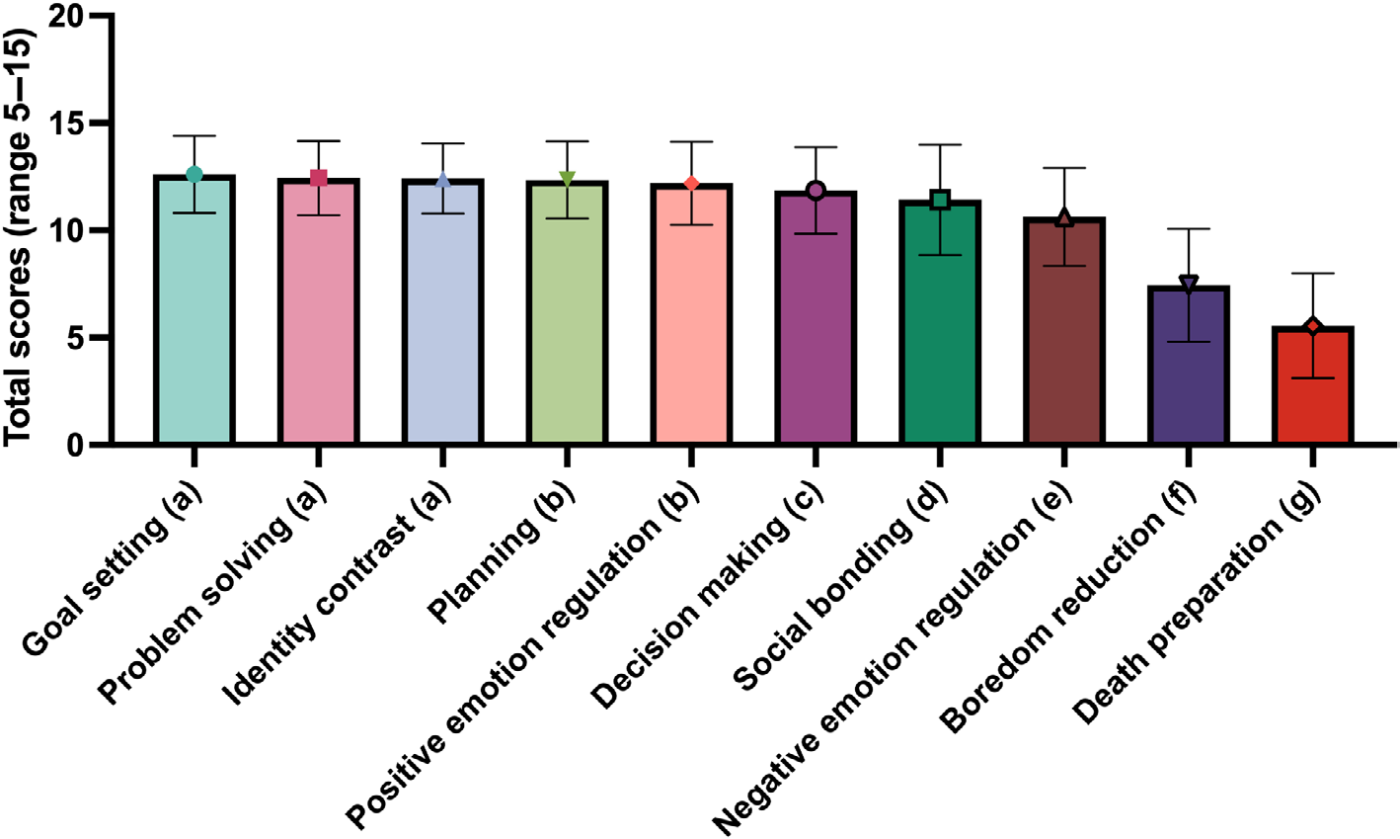
Total scores on Chinese FoFTS subscales. *Note:* Following Bonferroni corrections, subscales with different letters have significantly different means (all at least *p* < 0.05).

Figure 2 depicts the total score of each subscale of Chinese FoFTS in adults. The repeated ANOVA indicated significant differences among the 10 subscales (*F* (9,5193) = 970.74, *p* < 0.001, $\eta^{2}$ = 0.627). Pairwise comparisons revealed that the most frequent purposes of future thinking include goal setting, problem solving, and identity contrast, all without notable differences. The second most common functions are planning and positive emotion regulation, again with no significant differences. Following these are decision‐making, social bonding, negative emotion regulation, and boredom deduction, while the least common purpose identified was death preparation. The effect sizes indicating significant differences among the total scores of subscales ranged from small (*d* = 0.13, problem solving > positive emotion regulation) to large (*d* = 3.27, goal setting > death preparation).

### 3.7 Chinese FoFTS and Demographics

No sex differences were found in the functions of future thinking (all *p* values > 0.05). Education was negatively correlated with boredom reduction (*r* = −0.16, *p* < 0.001) and positively linked to problem solving (*r* = 0.11, *p* = 0.009), but not to any other functions of future thinking. Age was positively correlated with most functions (all *r* > 0.12, *p* < 0.001), except for boredom reduction, death preparation (both *r* < −0.10, *p* > 0.01), and negative emotion regulation (*r* = 0.08, *p* > 0.05).

Moreover, the results revealed age differences in FoFTS subscales (see Table 6). Middle‐aged adults reported a higher frequency of functions related to identity contrast, social bonding, goal setting, planning, problem solving, decision‐making, and positive emotion regulation than younger adults (all *p* values < 0.01). In contrast, both age groups showed similar functions in boredom reduction, death preparation, and negative emotion regulation (all *p* values > 0.05).

**TABLE 6:** Demographic and subscales characteristic of age groups.

Variables	Younger adults Mean/SD	Middle‐aged adults Mean/SD	Group differences
Sex: female (%)	53.71	48.47	*X* ^2^(1) = 1.58, *p* > 0.05
Age	25.48/3.29	37.88/7.48	*t* (576) = −25.62, *p* < 0.001
Years of Education	16.10/1.73	16.49/2.40	*t* (576) = −2.25, *p* = 0.024
FoFTS subscales			
Boredom	7.54/2.61	7.41/2.60	*F* (1, 575) = 0.06, *p* = 0.800
Death	5.75/2.57	5.40/2.32	*F* (1, 575) = 2.56, *p* = 0.110
Identity contrast	12.19/1.84	12.59/1.37	*F* (1, 575) = 7.76, *p* = 0.006
Negative emotion regulation	10.55/2.29	10.63/2.26	*F* (1, 575) = 0.12, *p* = 0.734
Social bonding	11.06/2.69	11.72/2.44	*F* (1, 575) = 8.80, *p* = 0.003
Goal setting	12.29/2.05	12.87/1.45	*F* (1, 575) = 14.16, *p* < 0.001
Planning	11.97/2.09	12.68/1.39	*F* (1, 575) = 22.19, *p* < 0.001
Problem solving	12.08/1.92	12.76/1.45	*F* (1, 575) = 21.04, *p* < 0.001
Decision making	11.46/2.22	12.19/1.72	*F* (1, 575) = 17.75, *p* < 0.001
Positive emotion regulation	11.83/2.22	12.51/1.58	*F* (1, 575) = 17.40, *p* < 0.001

### 3.8 Chinese FoFTS Across the Severity of Depression, Anxiety, and Stress

Table 7 shows that individuals experiencing moderate or above depression and stress exhibit significant differences in nearly all functions of future thinking, except for negative emotion regulation, when compared with those with normal or mild levels of depression and stress. In contrast, individuals experiencing moderate or above anxiety exhibited significant differences in all the functions of future thinking compared to those with normal or mild anxiety. Notably, those reporting higher levels of depression, anxiety, or stress scored significantly higher in boredom reduction and death preparation.

**TABLE 7:** A FoFTS‐based distinction across the levels of depression, anxiety, and stress.

Variables	Depression				ANCOVA		Effect‐size	Anxiety				ANCOVA		Effect‐size	Stress				ANCOVA		Effect‐size
	Normal or mild (*n* = 543)		Moderate or above (*n* = 35)					Normal or mild (*n* = 498)		Moderate or above (*n* = 80)					Normal or mild (*n* = 546)		Moderate or above (*n* = 32)				
	M	SD	M	SD	F	p	$\eta^{2}$	M	SD	M	SD	F	p	$\eta^{2}$	M	SD	M	SD	F	p	$\eta^{2}$
F1	7.39	2.57	8.80	2.74	7.33	**0.01**	0.01	7.31	2.60	8.53	2.41	9.82	**0.00**	0.02	7.38	2.57	9.19	2.67	12.27	**0.00**	0.02
F2	5.41	2.33	8.06	2.95	37.93	**0.00**	0.06	5.36	2.35	6.89	2.65	23.93	**0.00**	0.04	5.45	2.33	7.59	3.34	21.62	**0.00**	0.04
F3	12.55	1.44	10.03	2.41	82.77	**0.00**	0.13	12.59	1.43	11.21	2.20	43.63	**0.00**	0.07	12.50	1.50	10.63	2.50	37.51	**0.00**	0.06
F4	10.65	2.26	9.80	2.46	3.22	0.07	0.01	10.71	2.29	9.85	2.01	7.35	**0.01**	0.01	10.63	2.28	10.06	2.23	1.08	0.30	0.00
F5	11.59	2.43	8.49	3.17	42.81	**0.00**	0.07	11.73	2.36	9.35	2.98	51.17	**0.00**	0.08	11.56	2.44	8.66	3.37	33.71	**0.00**	0.06
F6	12.78	1.50	9.60	2.96	114.09	**0.00**	0.17	12.83	1.46	11.06	2.69	62.33	**0.00**	0.10	12.70	1.63	10.69	2.99	34.15	**0.00**	0.06
F7	12.46	1.66	10.29	2.61	42.98	**0.00**	0.07	12.60	1.54	10.64	2.33	78.11	**0.00**	0.12	12.45	1.67	10.38	2.66	35.15	**0.00**	0.06
F8	12.52	1.63	10.86	2.35	24.22	**0.00**	0.04	12.60	1.62	11.31	1.95	27.59	**0.00**	0.05	12.49	1.68	11.25	2.11	10.86	**0.00**	0.02
F9	11.94	1.95	10.26	2.37	16.76	**0.00**	0.03	12.05	1.89	10.46	2.23	32.25	**0.00**	0.05	11.91	1.97	10.50	2.24	10.15	**0.00**	0.02
F10	12.35	1.76	9.51	2.72	67.86	**0.00**	0.11	12.46	1.67	10.40	2.53	72.96	**0.00**	0.11	12.30	1.81	10.09	2.80	33.94	**0.00**	0.06

*Note:* Bold values are significant at *p* < 0.05 level. F1, boredom reduction; F2, death preparation; F3, identity contrast; F4, negative emotion regulation; F5, social bonding; F6, goal setting; F7, planning; F8, problem solving; F9, decision making; F10, positive emotion regulation.

## 4 Discussion

Thinking about the future is vital for human survival and reproduction. The inability to project oneself into the future can lead to functional consequences, such as short‐sighted decision‐making, lack of planning, and difficulties in social coordination (Suddendorf, Bulley, and Miloyan 2018). This study examined the psychometric properties and application of a Chinese version of self‐reported FoFTS, focusing on how future thinking serves the daily lives of Chinese community residents. The CFA results confirmed the 10‐factor structure of the Chinese version of FoFTS, aligning with prior studies (Akbari et al. 2023; Hallford and D'Argembeau 2021). The internal consistency results indicated that the Chinese version of FoFTS has an acceptable reliability. Therefore, the Chinese version of FoFTS demonstrated reasonable reliability and structure validity, indicating it is an effective scale for assessing the functions of future thinking within the Chinese context.

The findings of this study in Chinese adults are consistent with an earlier study involving Australian participants (Hallford and D'Argembeau 2021), which suggested that future thinking mainly serves directive‐type functions, such as goal setting, problem solving, planning, and decision making. Likewise, several studies have shown similar results, suggesting that the primary function of future thinking is to simulate current and potential scenarios, formulate intentions, and make future decisions (Branch and Zickar 2020; Schacter, Benoit, and Szpunar 2017; Szpunar, Spreng, and Schacter 2014). Notably, the study revealed that Chinese adults express different frequencies in the key functions of future thinking than their Australian counterparts. Specifically, Chinese adults reported that their most frequent functions of future thinking were goal setting, problem solving, and identity contrast. In contrast, Australian adults had the highest frequency in goal setting, planning, and decision‐making. It is noted that among Chinese adults, identity contrast occurs more often, whereas planning and decision‐making are less common. This may stem from the holistic thinking style often attributed to East Asians, who perceive ongoing changes and fluctuations (Choi, Koo, and Choi 2007). Holding a cyclical view, the Chinese tend to focus more on their future selves (identity contrast) because of the ever‐shifting environment. Since the objects change dramatically over time, they are less likely to engage in planning (planning) and make decisions for the future (decision making), thereby minimizing the time spent on unproductive activities. Further empirical studies directly comparing cultural differences will be necessary to enhance our understanding of how culture affects the functions of future thinking.

Results on convergent and incremental validity results revealed that functions of future thinking were widely correlated with time perspective, future self‐continuity, intertemporal decision making, and emotion regulation. The findings suggest that the capacity to vividly imagine future events necessitates a future time perspective. (D'Argembeau et al. 2010). Future self‐continuity was significantly predicted by positive emotion regulation, goal setting, identity contrast, decision making, social bonding, and boredom reduction, consistent with earlier research indicating that future self‐continuity can be enhanced by simulating future selves (Peters et al. 2010) and imagining more vivid positive future events (Zhang and Aggarwal 2015). Combined with earlier results revealing the effect of future thinking on future self‐continuity (Sun et al. 2023), the current study suggested a reciprocal relationship between future self‐continuity and episodic future thinking. Moreover, contrary to the finding that individuals who frequently engage in future thinking for planning tend to discount future rewards less (Eivazi et al. 2022), the current study suggested that the impact of future thinking on intertemporal decision making mainly depends on the content of imagined future events (Schacter, Benoit, and Szpunar 2017), such as the kind of person one will be, preparing for death, and the down‐regulation of negative emotions.

In this study, we applied the Chinese FoFTS in community adults to explore the effect of age on the functions of future thinking. Our findings provided insights into the relationship between age and the functions of future thinking. Specifically, we found that as people age, they tend to exhibit a greater frequency of functions of identity contrast, social bonding, goal setting, planning, problem solving, decision‐making, and positive emotion regulation. Moreover, our study revealed that younger and middle‐aged adults reported similar frequencies of boredom reduction, death preparation, and negative emotion regulation functions. Nonetheless, middle‐aged adults reported a higher frequency of frequent future thinking regarding planning, problem solving, decision‐making, goal setting, identity contrast, positive emotion regulation, and social bonding. This finding aligned with the life history theory, which posits that humans weigh trade‐offs between immediate and future reproduction (Williams 1957) and that middle‐aged adults tend to focus more on future‐oriented goals as they mature to maximize reproductive potential. Previous findings reported that middle‐aged adults have lower delay discounting than their younger counterparts (Lu et al. 2023), potentially reflecting a greater focus on future‐oriented thinking. Our study revealed key insights into how the functions of future thinking evolve as we age. This understanding can guide the development of targeted interventions to enhance future‐oriented behaviors across various age groups.

Furthermore, the results of the application of Chinese FoFTS across DASS suggested that planning is crucial for predicting reduced depression, anxiety, and stress, aligning with previous findings (Branch 2023). Conversely, boredom reduction and death preparation were found to increase depression, anxiety, and stress. Thinking more about task‐unrelated events and possible future death may heighten the risk of depressive thoughts and behaviors (Hallford and D'Argembeau 2021). Furthermore, identity contrast, goal setting, positive and negative emotion regulation, along with decision making, were identified as predictors of depression. Reduced negative emotion regulation and lack of future goals may result in repetitive, pessimistic thoughts about the future, potentially leading to depressive symptoms (Miranda et al. 2023). Fewer thoughts about the consequences or outcomes of behavior and less likely to imagine potential solutions for future issues and problems can cause increased feelings of depression (Hallford and D'Argembeau 2021). In contrast, stress was found to be predicted by social bonding. Future thinking was negatively correlated with stress. In patients with post‐traumatic stress disorder, future thinking showed a negative correlation with stress symptoms (Zlomuzica et al. 2018). Consequently, a diminished capacity for bonding with others might elevate stress levels. Additionally, this study showed that future thinking was correlated with emotion regulation, consistent with earlier research (Branch and Zickar 2020). Overall, these results indicate that the Chinese version of FoFTS is effective in differentiating between depression, anxiety, and stress, offering important insights and potential for personalized treatment and interventions.

This study has several limitations. First, we randomly recruited samples online, potentially restricting our sample to participants who regularly use smartphones and computers in their daily lives. Also, the current study did not include older adults aged 60 and above, limiting the generalizability of the findings. Future research should involve larger sample sizes and include older adults. Meanwhile, the FoFTS scale is a self‐report and retrospective measure of daily life. An ecological momentary assessment needs to be developed for this scale across studies. Finally, this study measured the self‐report depression, anxiety, and stress among general community samples, revealing differences in the FoFTS subscales. Future applications of this scale in various clinical samples could provide insights into how functions vary among clinical populations and link future thinking to maladaptive behaviors.

## 5 Conclusion

In conclusion, the validated Chinese version of FoFTS is psychometrically sound and can be used in Chinese samples to investigate the various purposes and functions of future thinking. Younger and middle‐aged adults showed different purposes for imagining future events. Additionally, it serves as an effective tool for differentiating individuals with varying levels of severity in depression, anxiety, and stress.

## Ethics Statement

The present study was approved by the ethics committee of University of Macau (SSHRE23‐APP068‐FSS).

## Conflicts of Interest

The authors declare no conflicts of interest.

## Data Availability

Data for this study is available by contacting the corresponding author upon reasonable request.
